# Supplementary material for: Astrocytes amplify neurovascular coupling to sustained activation of neocortex in awake mice
Source: Nat Commun. 2022 Dec 22;13:7872. doi: 10.1038/s41467-022-35383-2 (PMC9780254; doi:10.1038/s41467-022-35383-2)
Supplement: Supplementary file 1 — Supplementary Information [file 41467_2022_35383_MOESM1_ESM.pdf]

Supplementary Information, Institoris A et al.

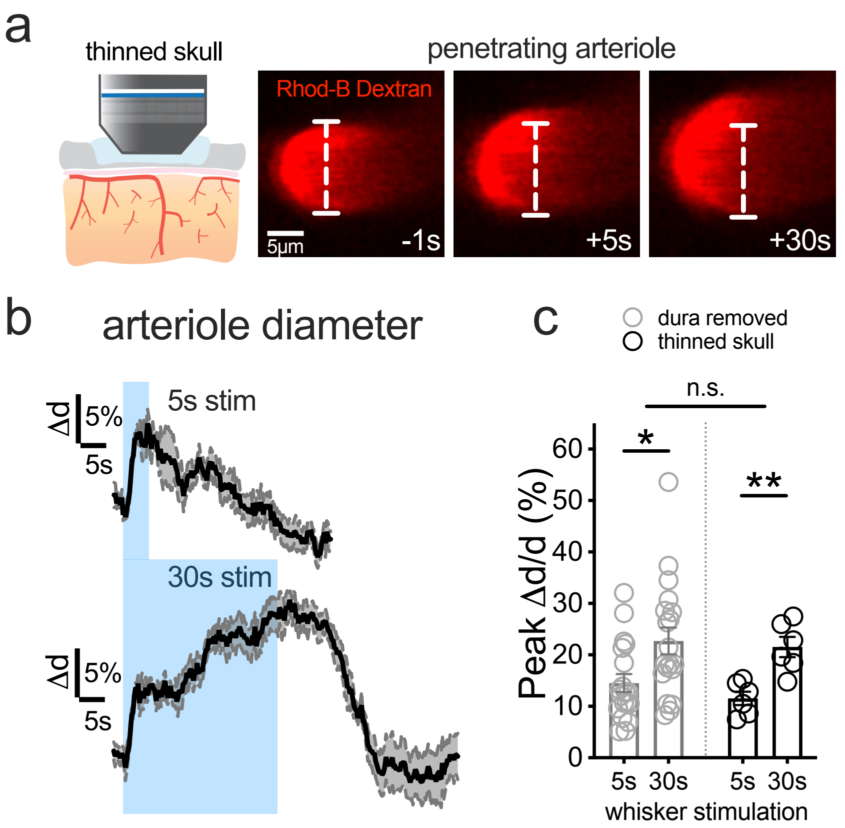

<u>Supplementary Figure</u> **1: The bimodal functional hyperemia response is preserved after dura removal. a**) Cartoon of the experimental preparation (*Left*) and 2-photon images of i.v. Rhodamine (Rhod)-B-dextran (red) labelled penetrating arteriole (PA) before and during 30sec whisker stimulation imaged through an acute thinned skull preparation (*Right*). Example of 6 experiments in 3 mice. **b**) Averaged traces of arteriole dilation to 5sec (upper) and 30sec (lower) whisker stimulation shows a bimodal arteriole response to 30sec stimulation in a thinned skull preparation. *N*= 6 PA (average of 18 trials) from 3 mice. Data shown are mean ± SEM. **c**) Summary data of peak diameter changes of PA (trials averaged) comparing responses of the thinned skull preparation (black) to the responses of dura removed preparation (grey). Dura removed: Paired *t* test (two-sided) $t(18)=2.46$; *$p=0.0242$. *N*=19 PA (average of 2-3 trials per PA) from 14 mice. Thinned skull: Paired *t* test (two-sided) $t(5)=5.02$; **$p=0.0040$. *N*= 6 PA (average of 1 8 trials) from 3 mice. Dura removed vs. thinned skull: Two-way ANOVA (two-sided). $F(1,23)=0.5130$, overall effect $p<0.4557$. Data shown are mean ± SEM. Source data are provided as a Source Data file.

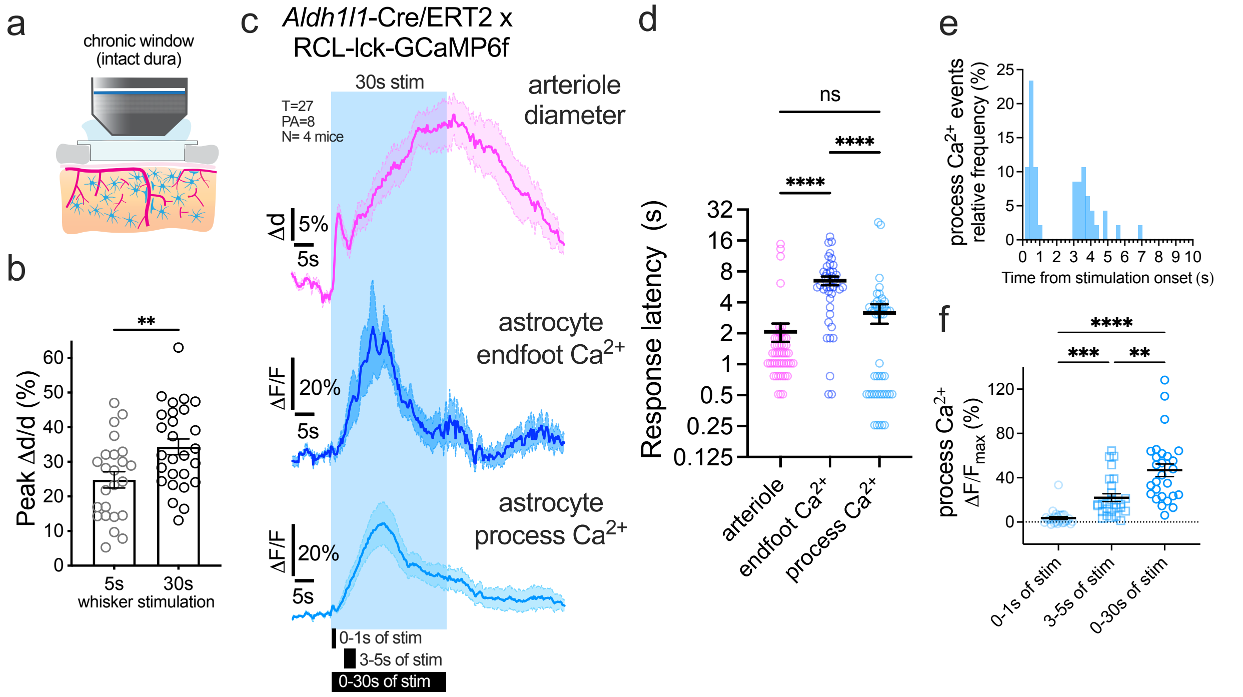

Supplementary Figure 2: **Astrocyte Ca^{2+} signals of membrane tethered GCaMP6f during sustained functional hyperemia.** **a**) Cartoon of the chronic cranial window preparation implanted with a T-shaped circular coverslip over the dura. **b**) Averaged traces of arteriole dilation to 5sec and 30sec whisker stimulation. Unpaired *t* test; *t*(49)=2.908; ***p*=0.0055. 5s: *n*=24 trials from 8 penetrating arterioles (PA), 30s: *n*=27 trials of 9 PA from 4 mice. Data shown are mean ± SEM. **c**) Arteriole diameter (magenta), astrocyte endfoot Ca^{2+} (dark blue) and fine process Ca^{2+} (light blue) responses (mean ± SEM) to 30sec whisker stimulation. *N*=9 PA or region of interest (ROI) (average of 3 trials each) from 4 mice. **d**) Summary of response onset (latency of signal > 3 x standard deviation above baseline) for dilation and astrocyte Ca^{2+}. Kruskal-Wallis test (one-sided) with Dunn's post hoc comparison. *H*(134)=39.26; overall effect: *****p*<0.0001. Arteriole vs endfoot Ca^{2+}: *****p*<0.0001, arteriole vs process Ca^{2+}: *p*>0.9999, endfoot Ca^{2+} vs process Ca^{2+}: *****p*<0.0001. 5sec and 30sec stimulations were pooled. Arteriole *n*= 50 trials, endfoot Ca^{2+}: *n*= 40 trials, process Ca^{2+}: *n*= 47 trials of 9 PA or ROI from 4 mice. All data are mean ± SEM. **e**) Relative frequency histogram of process Ca^{2+} events from stimulation onset reveals an ultrafast (0-1sec) and a delayed (3-5sec) population. **f**) The size of astrocyte process Ca^{2+} rise for ultrafast, delayed and overall (0-30sec) signals during stimulation. Friedman test (two-sided): *Q*(2)=52.63; *****p*<0.0001, with Dunn's post hoc comparison: 0-1s of stim vs 3-5s of stim: ****p*=0.0003, 0-1s of stim vs. 0-30s of stim: *****p*<0.0001, 3-5s of stim vs. 0-30s of stim: ***p*=0.0033. *N*= 27 trials of 9 ROI from 4 mice. All data are mean ± SEM. Source data are provided as a Source Data file.

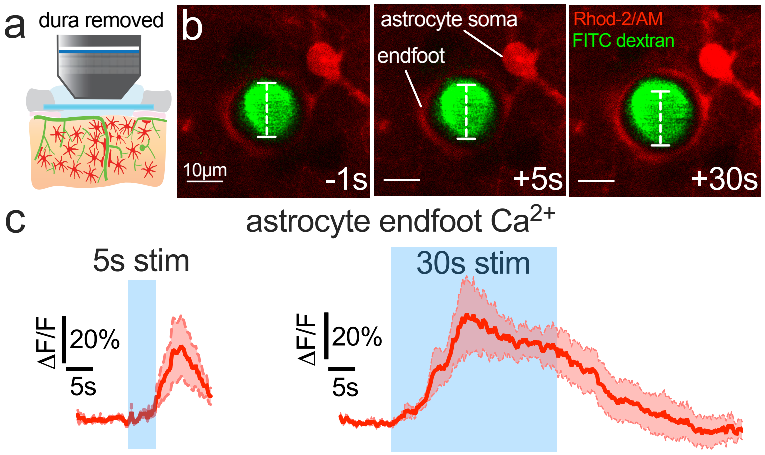

<u>Supplementary Figure 3.</u> Astrocyte endfoot Ca^{2+} signals of Rhod-2/AM loaded astrocytes. **a**) Representative cartoon of a dura-removed, fully sealed acute cranial window loaded with the Ca^{2+} indicator Rhod-2/AM. **b**) Time series images (top) of a Rhod-2/AM labelled astrocyte around a FITC-dextran (green) labelled penetrating arteriole. Example of 7 experiments from 5 mice. **c**) Astrocyte Ca^{2+} traces to 5sec (*Left*) and 30sec (*Right*) whisker stimulation. 5s stim: *n*=6 ROI (average of 3-4 trials) in 4 mice. 30s stim: *n*=7 ROI (average of 3-4 trials) in 5 mice. Data shown are mean ± SEM. Source data are provided as a Source Data file.

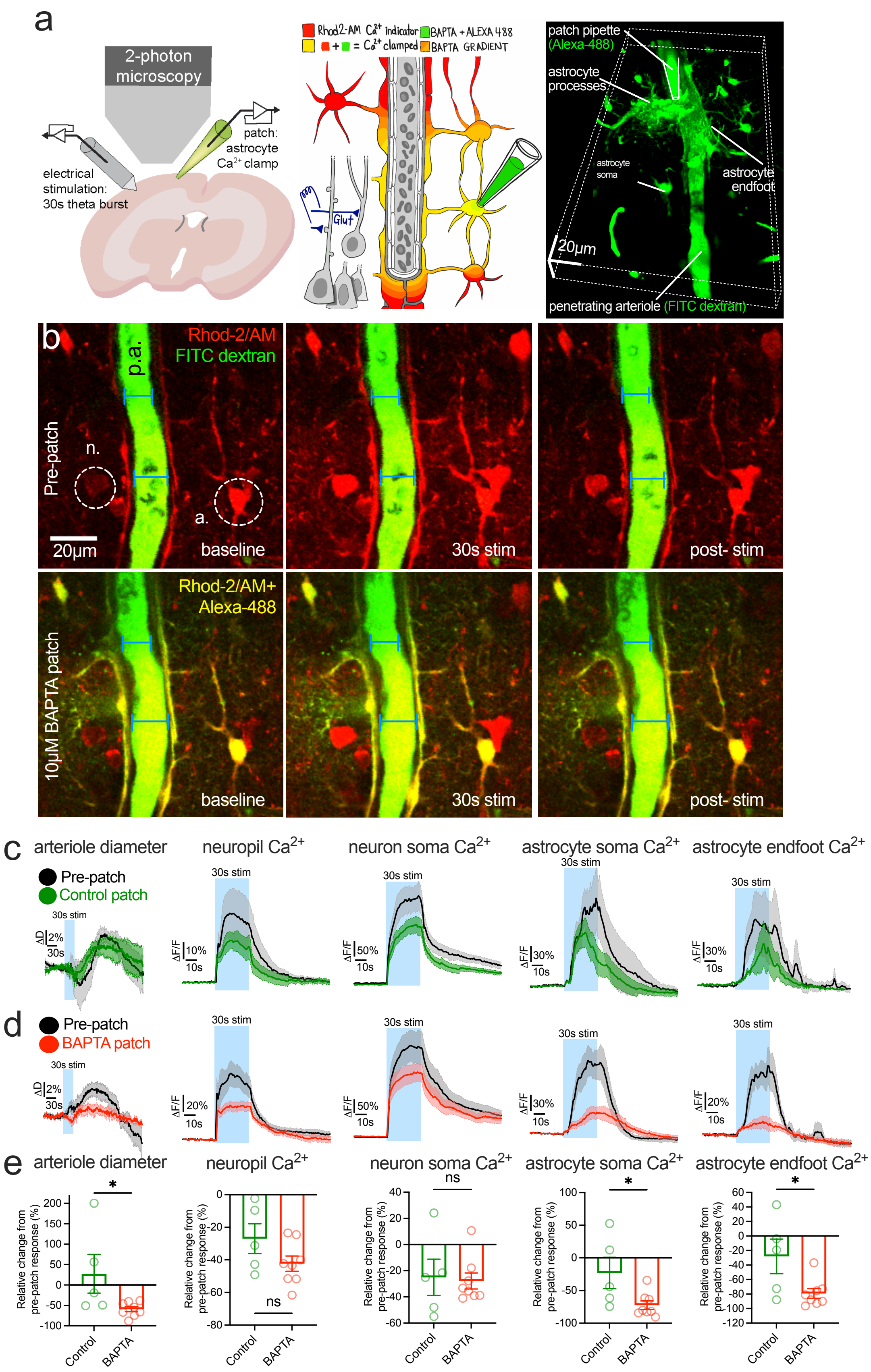
a
2-photon microscopy
electrical stimulation: 30s theta burst
patch: astrocyte Ca2+ clamp
Rhod2-AM Ca2+ indicator
BAPTA + ALEXA 488
Ca2+ clamped
BAPTA GRADIENT
Glut
patch pipette (Alexa-488)
astrocyte processes
astrocyte soma
astrocyte endfoot
20μm
penetrating arteriole (FITC dextran)
b
Rhod-2/AM
FITC dextran
p.a.
Pre-patch
n.
a.
20μm
baseline
30s stim
post- stim
Rhod-2/AM+ Alexa-488
10μM BAPTA patch
c
arteriole diameter
neuropil Ca2+
neuron soma Ca2+
astrocyte soma Ca2+
astrocyte endfoot Ca2+
Pre-patch
Control patch
30s stim
d
Pre-patch
BAPTA patch
e
Relative change from pre-patch response (%)
Control
BAPTA
ns
*

<u>Supplementary Figure 4</u>: Astrocyte Ca^{2+} clamp in brain slices with patched BAPTA reduces arteriole dilation to 30sec of high frequency afferent stimulation. **a**) *Left:* cartoon of experimental brain slice setup. *Middle:* patch infusion of BAPTA into the astrocyte network. *Right*: rotated z-stack of astrocytes patch-filled with Alexa-488 hydrazide (green) around a FITC-dextran labelled penetrating arteriole (PA) (green). Example of 13 slice experiments from 13 rats. **b**) *Upper:* Image time series showing astrocyte Ca^{2+} elevation and dilation to 30sec of theta burst electrical stimulation of afferents. Red: Rhod-2/AM labelled astrocytes (brighter) and neurons (fainter). Green: FITC-dextran labelled PA. *Lower:* the same stimulation is given in the presence of astrocyte network Ca^{2+} clamp (yellow astrocytes) and vasodilation is blocked. **c** and **d**) Average time series traces in response to 30 sec of afferent stimulation showing arteriole diameter, neuropil Ca^{2+}, neuron soma Ca^{2+}, astrocyte soma Ca^{2+} and endfoot Ca^{2+}. Control, pre-patch traces are shown (black), followed by a patch infusion of a control internal solution (upper green traces) or a Ca^{2+} clamp internal solution containing BAPTA (lower red traces). Control patch: n=5 slices from 5 rats. BAPTA patch: n=8 slices from 8 rats. Data shown are mean ± SEM. **e**) Summary data of percent changes from the pre-patch responses to either the control patch or the BAPTA patch condition. These data show that only the reduction in astrocyte Ca^{2+} can explain the loss of dilation to 30sec stimulation in the astrocyte BAPTA patch condition. Arteriole diameter: Unpaired t test (two-sided): $t(11)=2.331$; *p=0.0398. Neuropil Ca^{2+}: Unpaired t test (two-sided): $t(11)=1.662$; p=0.1247. Neuron soma Ca^{2+}: Mann-Whitney test (two-sided): U=20; p>0.9999. Astrocyte soma Ca^{2+}: Unpaired t test (two-sided): $t(11)=2.438$; *p=0.033. Astrocyte endfoot Ca^{2+}: Mann-Whitney test (two-sided): U=5; *p=0.0295. Control patch: n=5 slices from 5 rats. BAPTA patch: n=8 slices from 8 rats. Data shown are mean ± SEM. Source data are provided as a Source Data file.

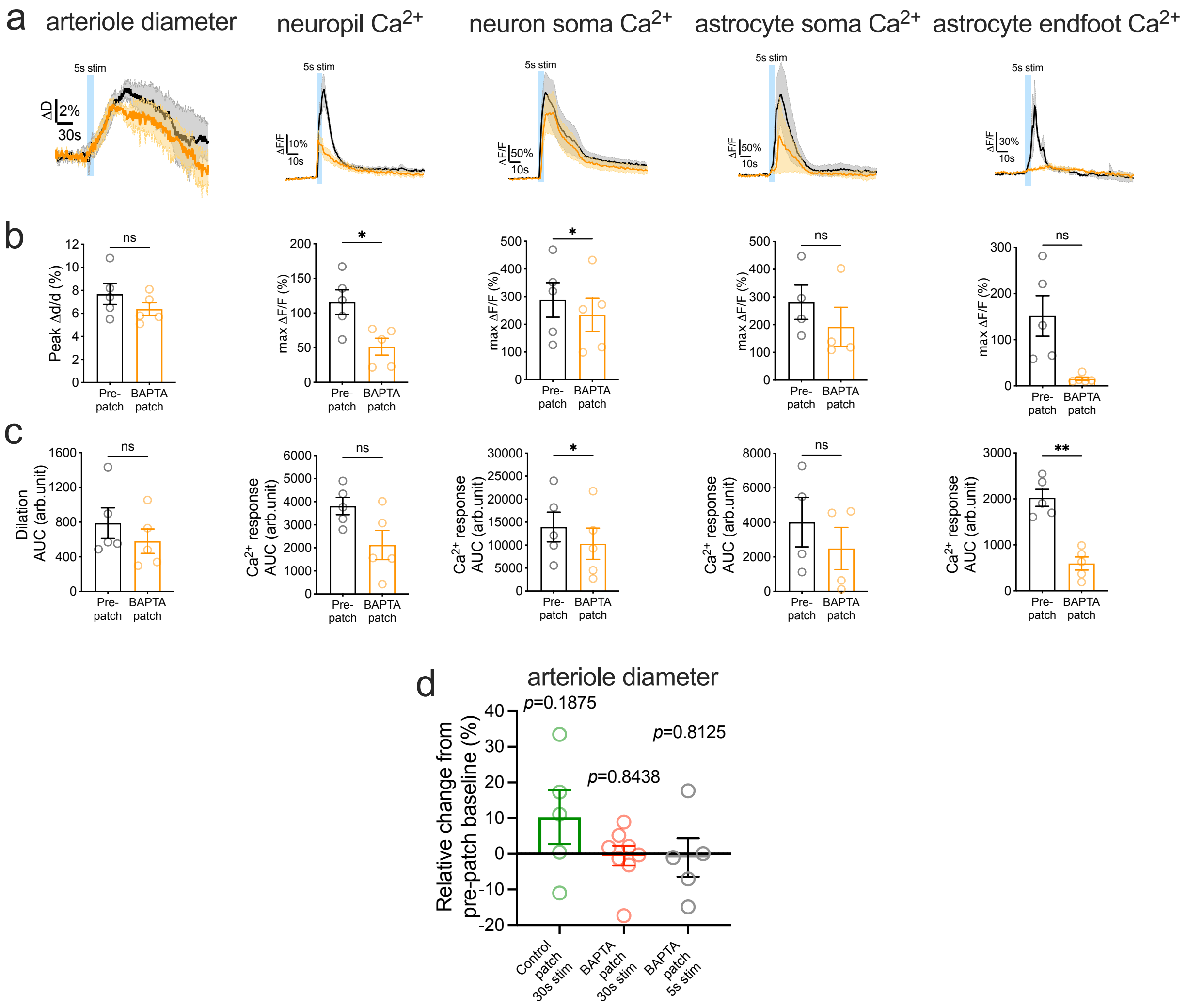

<u>Supplementary Figure 5:</u> Astrocyte Ca^{2+} clamp in brain slices with patched BAPTA has no effect on evoked arteriole dilation to 5sec high frequency afferent stimulation. **a**) Average time series traces (mean ± SEM) in response to 5sec of theta burst afferent stimulation in pre-astrocyte patch (black) or Ca^{2+} clamp patch (orange) showing arteriole diameter, neuropil Ca^{2+}, neuron soma Ca^{2+}, astrocyte soma Ca^{2+} and endfoot Ca^{2+}. *N*=5 slices from 5 rats. **b**) Summary data of peak responses (mean ± SEM). Peak Δd/d: Paired *t* test (two-sided): $t(4)=0.9802$; $p=0.3825$. Neuropil Ca^{2+} maxΔF/F: Paired *t* test (two-sided): $t(4)=2.942$; $*p=0.0423$. Neuron soma Ca^{2+} maxΔF/F: Paired *t* test (two-sided): $t(4)=3.450$; $*p=0.0261$. Astrocyte soma Ca^{2+} maxΔF/F: Wilcoxon test (two-sided): $W=-10$; $p=0.125$. Astrocyte endfoot Ca^{2+} max ΔF/F: Wilcoxon test (two-sided): $W=-15$; $p=0.0625$. **c**) Summary data of area under the curve (AUC). Dilation AUC (arbitrary(arb.) unit): Paired *t* test (two-sided): $t(4)=0.9802$; $p=0.3825$. Neuropil Ca^{2+} response AUC: Paired *t* test (two-sided): $t(4)=2.417$; $p=0.073$. Neuron soma Ca^{2+} response AUC: Paired *t* test (two-sided): $t(4)=3.345$; $*p=0.0287$. Astrocyte soma Ca^{2} response AUC: Paired *t* test (two-sided): $t(4)=3.079$; $p=0.0542$. Astrocyte endfoot Ca^{2+} response AUC: Paired *t* test (two-sided): $t(4)=5.584$; $**p=0.005$. *N*= 5 slices from 5 rats. Data are mean ± SEM. **d**) Summary data showing that neither the control patch internal solution, nor the Ca^{2+} clamp internal solution to 100nM free

Ca^{2+} (in the 5sec and 30sec experiments) affected resting arteriole diameter after the 15min whole-cell equilibration period. Pre-patch arteriole baseline is set as 0% for all 3 experiments. Pre-patch vs. Control patch 30s stim: Wilcoxon test (two-sided): W=11; p=0.1875 (n=5 slices from 5 rats), pre-patch vs. BAPTA patch 30s stim: Wilcoxon test (two-sided): W=4; p=0.8438 (n=8 slices from 8 rats), pre-patch vs. BAPTA patch 5s stim: Wilcoxon test (two-sided): W=-3; p=0.8125 (n=5 slices from 5 rats). Columns and error bars are mean ± SEM. Source data are provided as a Source Data file.

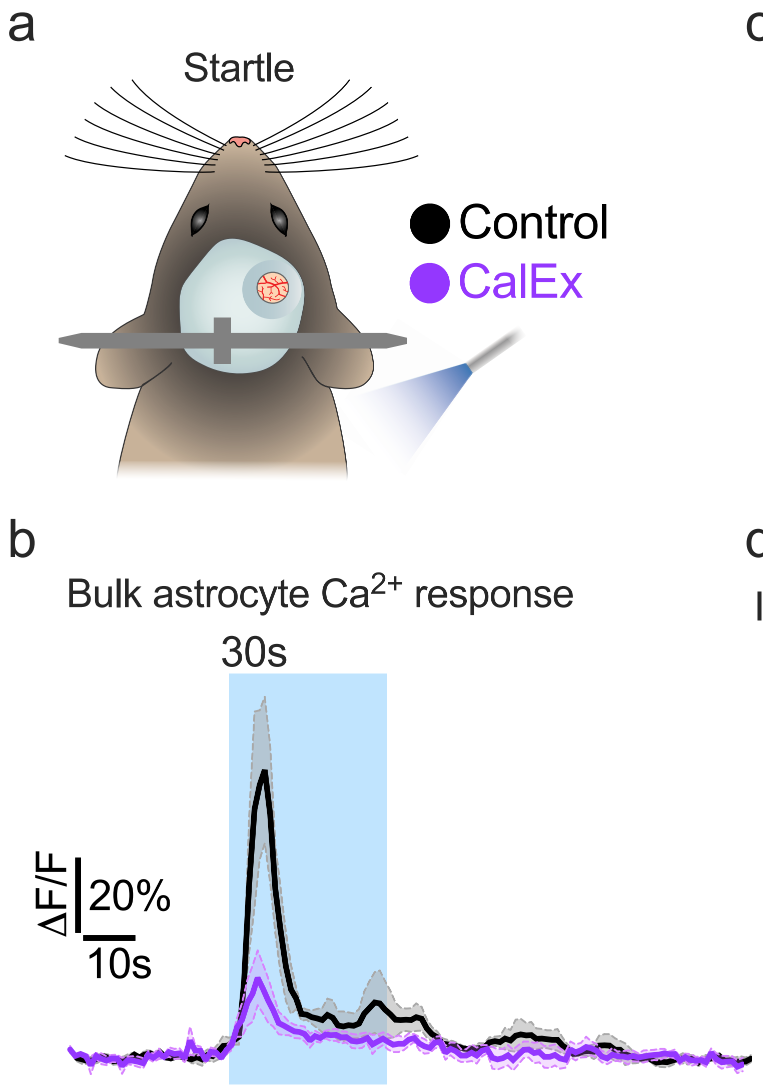

__Supplementary Figure 6__: **Expression of astrocytic plasma membrane Ca^{2+} ATPase (CalEx) decreases the evoked Ca^{2+} response to startle**. **a**) Cartoon of experimental setup using an untrained body air puff to startle the mouse. **b**) Average time series curves of astrocyte Ca^{2+} in response to startle, with CalEx and GCaMP6f AAV (purple) vs control AAVs (black). *N*=6 regions of interests (ROI) in 6 mice for both groups. Curves show mean ± SEM. **c**) Summary data of peak Ca^{2+} response (max ΔF/F%). Unpaired *t* test (two-sided): *t*(10)=2.555; *p=0.0286. Data shown are mean ± SEM. **d**) Summary data of integral Ca^{2+} response calculated as area under the curve (AUC). Unpaired *t* test (two-sided): *t*(10)=2.588; *p=0.0271. *N*=6 regions of interests (ROI) in 6 mice for both Control and CalEx groups in *c-d*. Data shown are mean ± SEM. Source data are provided as a Source Data file.

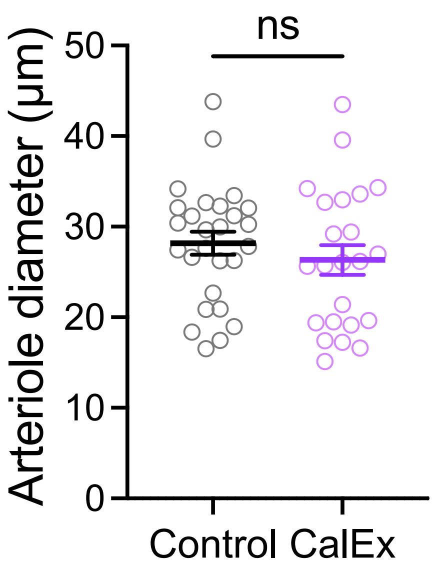

Supplementary Figure 7: CalEx did not change baseline penetrating arteriole (PA) diameter.

Baseline arteriole diameter of Control (*n*=27 PA from N=11 mice) and CalEx (*n*=23 PA from 10 mice) arterioles calculated from averaged 10sec pre-stimulus baseline recording (3-7 trials per PA). Unpaired *t* test (two-sided): $t(48)=0.9077$; $p=0.3686$. Data presented are mean ± SEM. Source data are provided as a Source Data file.

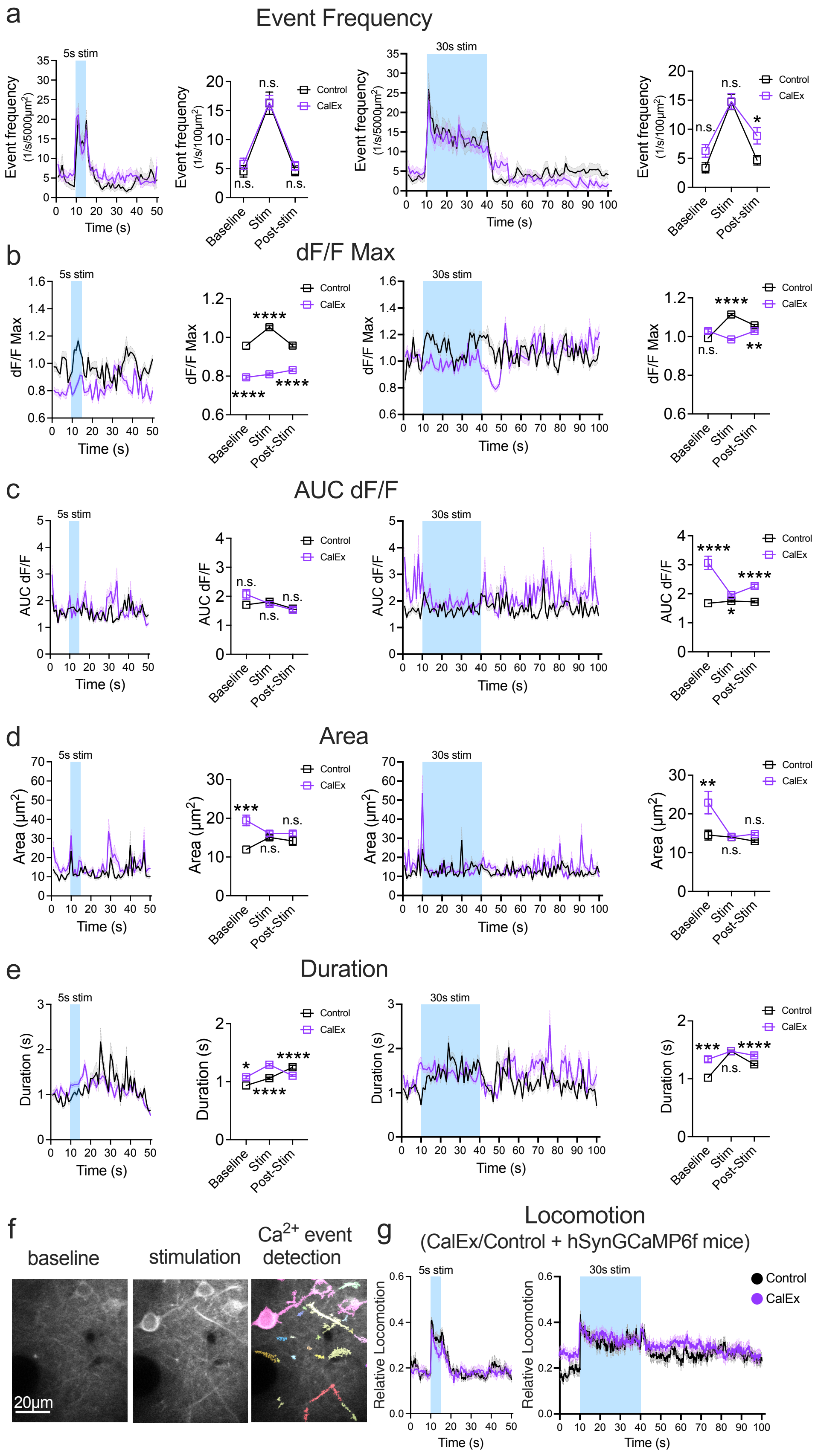
a
Event Frequency
5s stim
30s stim
Event frequency (1/s/5000µm²)
Event frequency (1/s/100µm²)
Time (s)
Control
CalEx
n.s.
*
Baseline
Stim
Post-stim
b
dF/F Max
****
**
c
AUC dF/F
*
d
Area
Area (µm²)
***
e
Duration
Duration (s)
f
baseline
stimulation
Ca2+ event detection
20µm
g
Locomotion
(CalEx/Control + hSynGCaMP6f mice)
Relative Locomotion

<u>Supplementary Figure 8</u>: Automated Ca^{2+} event detection analysis shows neuronal Ca^{2+} differences are unrelated to CalEx effect on arteriole. a) *Left:* Absolute neuronal (soma + neuropil) Ca^{2+} event frequency curves (1sec binning of 7.91Hz recording, mean ± SEM) Control (black), CalEx (purple) and averaged event frequencies of baseline, stimulation, and post-stimulation periods for 5sec whisker stimulation. Two-way repeated measures ANOVA with Sidak's multiple comparison test (two-sided). For 5s stim Control vs. CalEx comparison: $F(1,57)=0.4223$, overall effect $p=0.5184$. Baseline $p=0.7299$, Stim $p>0.9999$, Post-stim $p=0.842$. Control: $n=27$ trials of 10 experiments from 5 mice. CalEx: $n=32$ trials of 11 experiments from 5 mice. *Right:* Same but for 30sec stimulation. Two-way repeated measures ANOVA with Sidak's multiple comparison test (two-sided). For 30s stim Control vs. CalEx comparison: $F(1,61)=3.982$, overall effect $p=0.0505$. Baseline $p=0.1151$, Stim $p>0.9999$, Post-stim $^*p=0.0382$. Control: $n=28$ trials of 10 experiments from 5 mice. CalEx: $n=35$ trials of 11 experiments from 5 mice. Same layout for panels *b*-*e*. Data presented are mean ± SEM. **b**) Summary curves and averaged values of baseline, 5sec (*Left*) and 30sec (*Right*) stimulation and post-stimulation periods for maximal relative fluorescence of individual events (Max dF/F) show significantly larger Ca^{2+} peaks for control than for CalEx-injected mice but not during the later phase of 30sec stimulation. Two-way ANOVA with Tukey's multiple comparison test (two-sided). For 5s stim Control vs. CalEx comparison: $F(1,18721)=643.8$, overall effect $^{****}p<0.0001$. Baseline $^{****}p<0.0001$, Stim $^{****}p<0.0001$, Post-stim $^{****}p<0.0001$. Control: $n=27$ trials of 10 experiments from 5 mice. CalEx: $n=32$ trials,11 experiments from 5 mice. *Right:* Same but for 30sec stimulation. Two-way ANOVA with Tukey's multiple comparison test (two-sided). For 30s stim Control vs. CalEx comparison: $F(1,50412)=28.85$, overall effect $^{****}p<0.0001$. Baseline $p=0.5101$, Stim $^{****}p<0.0001$, Post-stim $^{**}p=0.0017$. Control: $n=28$ trials of 10 experiments from 5 mice. CalEx: $n=35$ trials of 11 experiments from 5 mice. **c**) Area Under the Curve (AUC) of individual neuronal Ca^{2+} event-related fluorescence changes (dF/F) demonstrate larger signals during baseline in the CalEx group. Two-way ANOVA with Tukey's multiple comparison test (two-sided). For 5s stim Control vs. CalEx comparison: $F(1,15444)=1.271$, overall effect $p=0.2595$. Baseline $p=0.1588$, Stim $p=0.9716$, Post-stim $p=0.9995$. Control: $n=27$ trials of 10 experiments from 5 mice. CalEx: $n=32$ trials of 11 experiments from 5 mice. *Right:* Same but for 30sec stimulation. Two-way ANOVA with Tukey's multiple comparison test (two-sided). For 30s stim Control vs. CalEx comparison: $F(1,50412)=91.79$, overall effect $^{****}p<0.0001$. Baseline $^{****}p<0.0001$, Stim $^*p=0.0111$, Post-stim $^{****}p<0.0001$. Control: $n=28$ trials of 10 experiments from 5 mice. CalEx: $n=35$ trials of 11 experiments from 5 mice. **d**) Area (size) of individual neuronal Ca^{2+} events are also larger at baseline for CalEx than control. Two-way ANOVA with Tukey's multiple comparison test (two-sided). For 5s stim Control vs. CalEx comparison: $F(1,18721)=20.31$, overall effect $^{****}p<0.0001$. Baseline $p=0.0002$, Stim $p=0.9452$, Post-stim $p=0.4596$. Control: $n=27$ trials of 10 experiments from 5 mice. CalEx: $n=32$ trials of 11 experiments from 5 mice. *Right:* Same but for 30sec stimulation. Two-way ANOVA with Tukey's multiple comparison test (two-sided). For 30s stim Control vs. CalEx comparison: $F(1,50412)=18.17$, overall effect $^{****}p<0.0001$. Baseline $^{**}p<0.0016$, Stim $^*p>0.9999$, Post-stim $p<0.2225$. Control: $n=28$ trials of 10 experiments from 5 mice. CalEx: $n=35$ trials of 11 experiments from 5 mice. **e**) The average duration of Ca^{2+} events for CalEx-injected mice are overall longer than for control virus injected mice except during sustained stimulation. Two-way ANOVA with Tukey's multiple comparison test (two-sided). For 5s stim Control vs. CalEx comparison: $F(1,18721)=12.92$, overall effect $^{***}p=0.0003$. Baseline $p=0.0377$, Stim $^{****}p<0.0001$, Post-stim $^{****}p<0.0001$. Control: $n=27$ trials of 10 experiments from 5 mice. CalEx: $n=32$ trials of 11 experiments from 5 mice. *Right:* Same but for 30sec stimulation. Two-way ANOVA with Tukey's multiple comparison test (two-sided). For 30s stim Control vs.

CalEx comparison: $F(1,50412)=29.04$, overall effect ****$p<0.0001$. Baseline ***$p<0.0008$, Stim *$p>0.9999$, Post-stim ****$p<0.0001$. Control: $n=28$ trials of 10 experiments from 5 mice. CalEx: $n=35$ trials of 11 experiments from 5 mice. All data in panels *a-e* are mean ± SEM. **f**) Raw 2-photon image of neuronal Ca^{2+} events in GCaMP6f expressing neuronal structures in layer 2 of the barrel cortex (*Left*) before and (*Middle*) during whisker stimulation. *Right*: Colour-coded detection of individual Ca^{2+} events by an automated Ca^{2+} event detection toolkit (https://github.com/yu-lab-vt/AQuA). **g**) Summary of relative locomotion curves for 5sec (*Left*) and 30sec (*Right*) whisker stimulation in mice injected with CalEx or its mutant control virus mixed with an AAV-hSynGCaMP6f virus indicate similar locomotion pattern during 30sec stimulation. Locomotion differences at baseline and 5sec stimulation between control and CalEx could account for the differences in individual Ca^{2+} event properties. Control 5sec: $n=28$ trials, 30sec: $n=30$ trials in 5 mice. CalEx 5sec: $n=31$ trials, 30sec: $n=37$ trials in 5 mice. Traces are mean ± SEM. Source data are provided as a Source Data file.

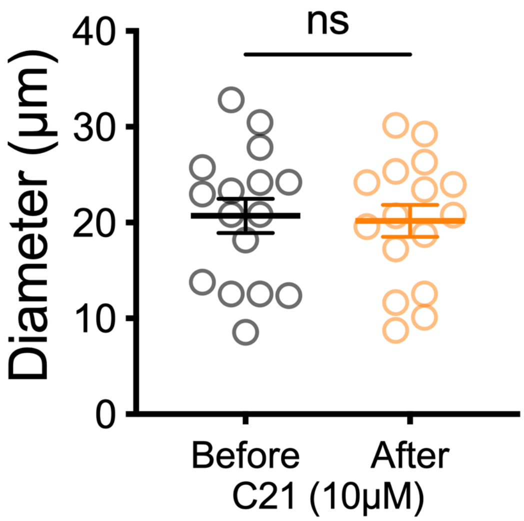

<u>Supplementary Figure 9</u>: Arteriole baseline diameter is not different before and after 1 hour of continuous astrocyte Gq receptor activation with C21. Baseline arteriole diameter before and after C21 application was calculated from averaged 10sec pre-stimulus baseline recording (*n*=16 penetrating arterioles (PA) from 6 mice, 2-3 trials per PA). Paired *t* test (two-sided): $t(15)=0.3219$; $p=0.752$. Data presented are mean ± SEM. Source data are provided as a Source Data file.

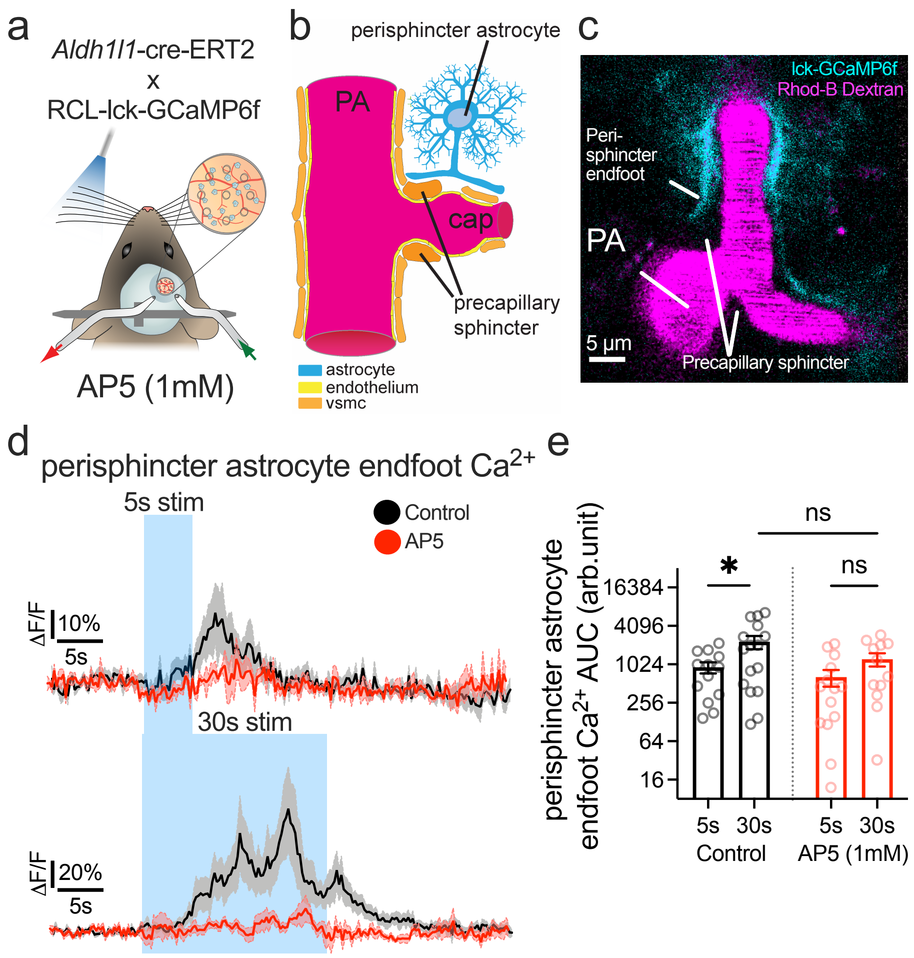

<u>Supplementary Figure 10</u>: Peri-sphincter astrocyte Ca^{2+} in response to 5sec and 30sec whisker stimulation. **a**) Cartoon of *in vivo* experimental setup using membrane tethered GCaMP6f in astrocytes. **b**) Cartoon depicting astrocyte of interest (blue), adjacent to a pre-capillary sphincter. Vsmc: vascular smooth muscle cell, cap: capillary, PA: penetrating arteriole. **c**) 2-photon image of a PA (magenta, median filtered) and a narrowing at the first branch off the penetrator where mural sphincter cells are located. Surrounding astrocytes expressing membrane targeted lck-GCaMP6f (blue) are shown. Example of experiments from 6 mice. **d**) Average time series trace data of astrocyte endfoot Ca^{2+} in pre-drug control (black) and in the presence of AP5 (red) surrounding an arteriole sphincter in response to 5sec (*n*=13 trials at 5 PA from 5 mice) or 30sec (*n*=16 trials at 6 PA from 6 mice) whisker stimulation. **e**) *S*ummary data of perisphincter astrocyte endfoot Ca^{2+} area under the curve (AUC). Two-way ANOVA with Tukey's multiple comparisons test (two-sided). For stimulation length comparison, $F(1,51)=7.270$, overall effect $^{**}p<0.0095$; Control (5s vs. 30s) $^{*}p<0.0396$, AP5 (5s vs. 30s) $p=0.688$. For AP5 treatment comparison, $F(1,51)=3.366$, overall effect $p<0.0724$; 5s (Control vs. AP5) $p=0.9533$, 30s (Control vs. AP5) $p=0.1737$. Interaction between stimulation length and AP5 treatment $F(1,51)=1.195$, overall effect $p<0.2795$. Data are shown as mean ± SEM. Source data are provided as a Source Data file.

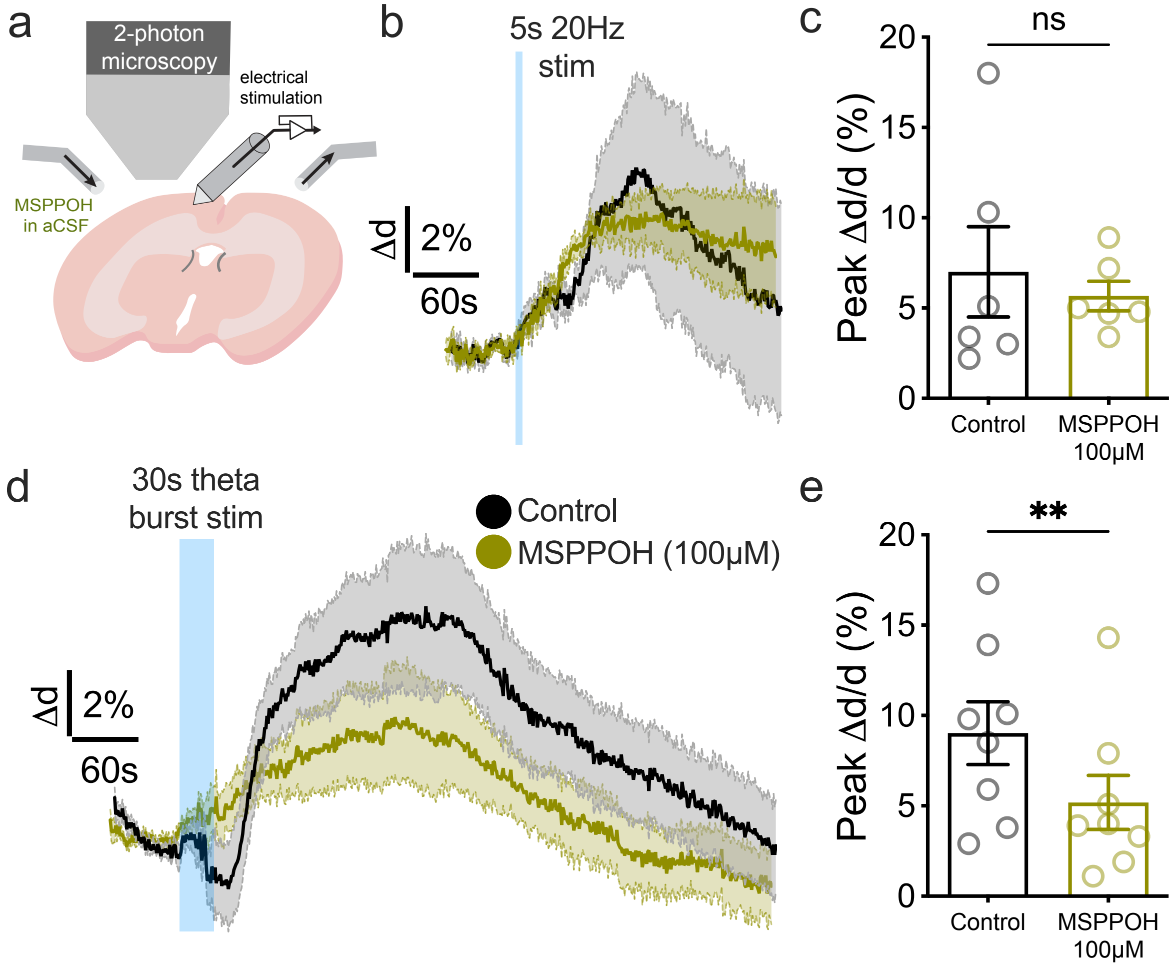

<u>Supplementary Figure 11</u>: Epoxygenase inhibition with MSPPOH reduces arteriole dilation to 30sec high frequency afferent stimulation but not to 5sec. **a**) Cartoon of experimental brain slice setup with electrical afferent stimulation. ACSF: artificial cerebrospinal fluid. **b**) Average ± SEM traces of evoked arteriole dilation to 5sec stim in pre-drug control (black) and in the presence of MSPPOH (green). **c**) Summary data (mean ± SEM) for 5sec stim, showing no effect of MSPPOH on peak arteriole diameter change (Δd/d%). Paired *t* test (two-sided). *t*(5)=0.7638; *p*=0.4794. *N*=6 slices from 5 rats. **d**) Average ± SEM traces of evoked arteriole dilation to 30sec electrical stim in pre-drug control (black) and in the presence of MSPPOH (green). **e**) Summary data (mean ± SEM) for 30sec stim, showing a significant reduction in peak arteriole diameter change (Δd/d%) by MSPPOH. Paired *t* test (two-sided). *t*(7)=3.521; ***p*=0.0097. *N*=8 slices from 7 rats. Source data are provided as a Source Data file.

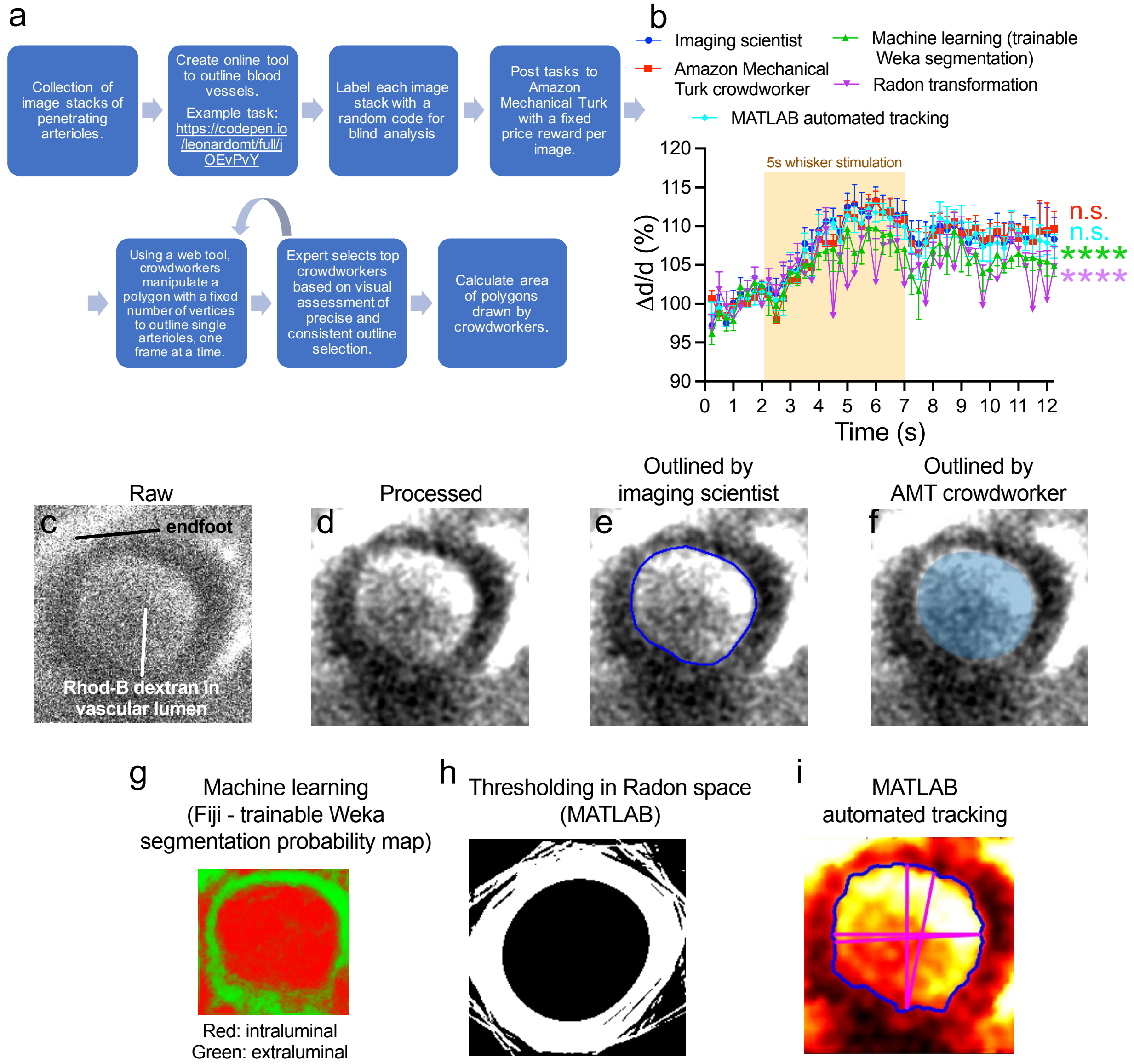

<u>**Supplementary Figure 12**</u>**: Validation of crowd sourced analysis of arteriole diameter changes**. **a**) Workflow of analysis using Amazon Turk with validation by imaging scientist. **b**) Analysis of arteriole diameter changes by 'trained' crowd-workers sourced via Amazon Turk (red) and MATLAB automated tracking (cyan) performed equally well as a trained imaging scientist (blue). Both these analyses outperformed an ImageJ machine learning tool called WEKA (green) as well as implementing a radon transform of the data (purple) (PMID 24736890). Friedman's test with Dunn's multiple comparison test. $F(4)=36.71$, overall significance ****$p<0.0001$. Imaging scientist vs. machine learning: ****$p<0.0001$, imaging scientist vs. Radon transform: ****$p<0.0001$ (purple), imaging scientist vs. Amazon Turk: $p=0.2946$ (red n.s.), imaging scientist vs. MATLAB automated tracking: $p=33.81$ (cyan n.s.). $N=5$ trials of 5 arterioles from 4 mice. Data presented are mean + or – SEM. **c-i**) representative images of arteriole lumen pre-processing ***(c,d)*** followed by the identification of the arteriole lumen by either an imaging scientist ***(e)*** or a crowd-worker ***(f)***, WEKA segmentation ***(g)***, thresholding in Radon Space ***(h)***, and MATLAB automated tracking based on Thirion's DEMONS algorithm (***i***). Source data are provided as a Source Data file.

Supplementary Table 1: Details of statistical analysis related to Figure 1.

Figure 1d

Friedman test (one-sided)		
Q	Summary	P value
11.14	**	0.0012
Dunn's multiple comparisons test		
Group comparisons	Summary	Adjusted P Value
1s vs. 5s	ns	>0.9999
1s vs. 30s	**	0.004
5s vs. 30s	*	0.0485

Figure 1e

Mixed effects model (Regression Model)		
F (DFn, DFd)	Summary	P value
F (1.481, 40.74) = 17.21	****	<0.0001
Holm-Sidak’s multiple comparison test		
Group comparisons	Summary	Adjusted P Value
arteriole dilation vs. astrocyte endfoot Ca^{2+}	****	<0.0001
arteriole dilation vs. astrocyte process Ca^{2+}	*	0.0118
astrocyte endfoot Ca^{2+} vs. astrocyte process Ca^{2+}	*	0.0118

Supplementary Table 2: Details of statistical analysis related to Figure 2.

Figure 2g

**Peak arteriole dilation**		
ANOVA table	F (DFn, DFd)	P value
Interaction	F (1, 96) = 5.992	P=0.0162
Stimulation length	F (1, 96) = 32.32	P<0.0001
CalEx	F (1, 96) = 5.713	P=0.0188
Tukey's multiple comparisons test	Summary	Adjusted P Value
5s:Control vs. 5s:CalEx	ns	>0.9999
5s:Control vs. 30s:Control	****	<0.0001
5s:Control vs. 30s:CalEx	ns	0.1028
5s:CalEx vs. 30s:Control	****	<0.0001
5s:CalEx vs. 30s:CalEx	ns	0.1296
30s:Control vs. 30s:CalEx	**	0.0046

**net Area Under the Curve of arteriole dilation**		
ANOVA table	F (DFn, DFd)	P value
Interaction	F (1, 96) = 3.514	P=0.063884
Stimulation length	F (1, 96) = 163.4	P<0.000001
CalEx	F (1, 96) = 11.03	P=0.001270
Tukey's multiple comparisons test	Summary	Adjusted P Value
5s:Control vs. 5s:CalEx	ns	0.741596
5s:Control vs. 30s:Control	****	<0.000001
5s:Control vs. 30s:CalEx	****	<0.000001
5s:CalEx vs. 30s:Control	****	<0.000001
5s:CalEx vs. 30s:CalEx	****	<0.000001
30s:Control vs. 30s:CalEx	**	0.001971

Figure 2j

Peak astrocyte endfoot Ca^{2+}		
ANOVA table	F (DFn, DFd)	P value
Interaction	F (1, 178) = 13.70	P=0.0003
Stimulation length	F (1, 178) = 20.93	P<0.0001
CalEx	F (1, 178) = 34.54	P<0.0001
Tukey's multiple comparisons test	Summary	Adjusted P Value
5s:Control vs. 5s:CalEx	ns	0.434
5s:Control vs. 30s:Control	****	<0.0001
5s:Control vs. 30s:CalEx	ns	0.7956
5s:CalEx vs. 30s:Control	****	<0.0001
5s:CalEx vs. 30s:CalEx	ns	0.9416
30s:Control vs. 30s:CalEx	****	<0.0001

net Area Under the Curve of astrocyte endfoot Ca^{2+}		
ANOVA table	F (DFn, DFd)	P value
Interaction	F (1, 178) = 12.07	P=0.0006
Stimulation length	F (1, 178) = 19.91	P<0.0001
CalEx	F (1, 178) = 16.66	P<0.0001
Tukey's multiple comparisons test	Summary	Adjusted P Value
5s:Control vs. 5s:CalEx	ns	0.9748
5s:Control vs. 30s:Control	****	<0.0001
5s:Control vs. 30s:CalEx	ns	0.9932
5s:CalEx vs. 30s:Control	****	<0.0001
5s:CalEx vs. 30s:CalEx	ns	0.9184
30s:Control vs. 30s:CalEx	****	<0.0001

Figure 2m

Peak astrocyte process Ca^{2+}		
ANOVA table	F (DFn, DFd)	P value
Interaction	F (1, 174) = 7.026	P=0.0088
Stimulation length	F (1, 174) = 14.31	P=0.0002
CalEx	F (1, 174) = 19.31	P<0.0001
Tukey's multiple comparisons test	Summary	Adjusted P Value
5s:Control vs. 5s:CalEx	ns	0.623
5s:Control vs. 30s:Control	****	<0.0001
5s:Control vs. 30s:CalEx	ns	0.9738
5s:CalEx vs. 30s:Control	****	<0.0001
5s:CalEx vs. 30s:CalEx	ns	0.8815
30s:Control vs. 30s:CalEx	****	<0.0001

net Area Under the Curve of astrocyte process Ca^{2+}		
ANOVA table	F (DFn, DFd)	P value
Interaction	F (1, 171) = 10.08	P=0.0018
Stimulation length	F (1, 171) = 8.443	P=0.0041
CalEx	F (1, 171) = 21.16	P<0.0001
Tukey's multiple comparisons test	Summary	Adjusted P Value
5s:Control vs. 5s:CalEx	ns	0.7539
5s:Control vs. 30s:Control	****	<0.0001
5s:Control vs. 30s:CalEx	ns	0.6349
5s:CalEx vs. 30s:Control	****	<0.0001
5s:CalEx vs. 30s:CalEx	ns	0.9981
30s:Control vs. 30s:CalEx	****	<0.0001

Figure 2p

Peak neuronal Ca^{2+}		
ANOVA table	F (DFn, DFd)	P value
Interaction	F (1, 126) = 0.3577	P=0.5508
Stimulation length	F (1, 126) = 14.27	P=0.0002
CalEx	F (1, 126) = 1.382	P=0.2420
Tukey's multiple comparisons test	Summary	Adjusted P Value
5s:Control vs. 5s:CalEx	ns	0.7765
5s:Control vs. 30s:Control	ns	0.1766
5s:Control vs. 30s:CalEx	ns	0.3222
5s:CalEx vs. 30s:Control	**	0.0048
5s:CalEx vs. 30s:CalEx	**	0.0099
30s:Control vs. 30s:CalEx	ns	0.9989

net Area Under the Curve of neuronal Ca^{2+}		
ANOVA table	F (DFn, DFd)	P value
Interaction	F (1, 126) = 0.7913	P=0.3754
Stimulation length	F (1, 126) = 32.60	P<0.0001
CalEx	F (1, 126) = 0.8580	P=0.3561
Tukey's multiple comparisons test	Summary	Adjusted P Value
5s:Control vs. 5s:CalEx	ns	>0.9999
5s:Control vs. 30s:Control	****	<0.0001
5s:Control vs. 30s:CalEx	**	0.0046
5s:CalEx vs. 30s:Control	****	<0.0001
5s:CalEx vs. 30s:CalEx	**	0.0034
30s:Control vs. 30s:CalEx	ns	0.7227

Supplementary Table 3: Details of statistical analysis related to Figure 3 (top 3 tables) and details of statistical analysis for locomotion co-variate analysis in a general linear model for variables presented in Figure 2 (arteriole peak $\Delta d/d$, net Area Under the Curve of arteriole dilation, neuronal Ca^{2+} max $\Delta F/F$, neuronal Ca^{2+} AUC, astrocyte endfoot Ca^{2+} max $\Delta F/F$, astrocyte endfoot Ca^{2+} AUC, astrocyte process Ca^{2+} max $\Delta F/F$, astrocyte process Ca^{2+} AUC)(bottom table).

Fig 3a

Mann Whitney test (two-tailed)	Relative Locomotion during 5s whisker stimulation
U Value	2137
P value	0.1991
P value summary	ns

Fig 3b

Mann Whitney test (two-tailed)	Relative Locomotion during 30s whisker stimulation
U Value	2555
P value	0.8977
P value summary	ns

Fig 3g

One-Way ANOVA		
F (DFn, DFd)	Summary	P value
F (2, 30) = 4.232	*	P=0.0240
Tukey's multiple comparisons test		
Group comparisons	Summary	Adjusted P Value
No stim vs. Startle	*	0.0218
No stim vs. Whisker stim	ns	0.0667
Startle vs. Whisker stim	ns	0.6826

Co-variate analysis of locomotion in a general linear model of CalEx and Control groups				
	df	F	p	Summary
arteriole peak Δd/d	278	0.48	0.489	ns
arteriole dilation AUC	278	0.56	0.813	ns
neuronal Ca^{2+} max ΔF/F	122	0.343	0.559	ns
neuronal Ca^{2+} AUC	122	0.12	0.912	ns
astrocyte process Ca^{2+} max ΔF/F	161	1.998	0.16	ns
astrocyte process Ca^{2+} AUC	161	0.237	0.627	ns
astrocyte endfoot Ca^{2+} max ΔF/F	161	10.699	0.001	**
astrocyte endfoot Ca^{2+} AUC	161	5.561	0.02	*

Supplementary Table 4: Details of statistical analysis related to Figure 4.

Figure 4d

Paired t test (two-tailed)	**Astrocyte soma Ca^{2+}**	**Astrocyte endfoot Ca^{2+}**	**Arteriole diameter**
P value	<0.0001	0.0002	<0.0001
P value summary	****	****	****
t, df	t=7.539, df=13	t=5.210, df=13	t=7.453, df=13

Figure 4e

**5s whisker stimulation**			
Peak dilation (%)		Dilation AUC	
Paired t test (two-tailed)		Paired t test (two-tailed)	
P value	0.0533	P value	0.0944
P value summary	ns	P value summary	ns
t, df	t=2.098, df=15	t, df	t=1.786, df=15

Figure 4f

**30s whisker stimulation**			
Peak dilation (%)		Dilation AUC	
Paired t test (two-tailed)		Paired t test (two-tailed)	
P value	0.0056	P value	0.0099
P value summary	**	P value summary	**
t, df	t=3.228, df=15	t, df	t=2.950, df=15

Figure 4i

**30s whisker stimulation**	
Peak neuronal Ca^{2+}	
Paired t test (two-tailed)	
P value	0.0056
P value summary	**
t, df	t=3.228, df=15

Supplementary Table 5: Details of statistical analysis related to Figure 5.

Figure 5c

Area Under the Curve of astrocyte endfoot Ca^{2+}		
ANOVA table	F (DFn, DFd)	P value
Interaction	F (1, 77) = 2.543	P=0.1149
Stimulation length	F (1, 77) = 6.838	P=0.0107
AP5	F (1, 77) = 9.712	P=0.0026
Tukey's multiple comparisons test	Summary	Adjusted P Value
5s:Control vs. 5s:AP5	ns	0.7362
5s:Control vs. 30s:Control	*	0.0161
5s:Control vs. 30s:AP5	ns	0.9856
5s:AP5 vs. 30s:Control	***	0.0005
5s:AP5 vs. 30s:AP5	ns	0.8952
30s:Control vs. 30s:AP5	**	0.004

Figure 5d

net Area Under the Curve of astrocyte process Ca^{2+}		
ANOVA table	F (DFn, DFd)	P value
Interaction	F (1, 96) = 3.514	P=0.063884
Stimulation length	F (1, 96) = 163.4	P<0.000001
AP5	F (1, 96) = 11.03	P=0.001270
Tukey's multiple comparisons test	Summary	Adjusted P Value
5s:Control vs. 5s:AP5	ns	0.741596
5s:Control vs. 30s:Control	****	<0.000001
5s:Control vs. 30s:AP5	****	<0.000001
5s:AP5 vs. 30s:Control	****	<0.000001
5s:AP5 vs. 30s:AP5	****	<0.000001
30s:Control vs. 30s:AP5	**	0.001971

Figure 5f

Peak arteriole dilation		
ANOVA table	F (DFn, DFd)	P value
Interaction	F (1, 28) = 3.080	P=0.0902
Stimulation length	F (1, 28) = 0.5535	P=0.4631
AP5	F (1, 28) = 6.919	P=0.0137
Tukey's multiple comparisons test	Summary	Adjusted P Value
5s:Control vs. 5s:AP5	ns	0.9251
5s:Control vs. 30s:Control	ns	0.3099
5s:Control vs. 30s:AP5	ns	0.5499
5s:AP5 vs. 30s:Control	ns	0.1032
5s:AP5 vs. 30s:AP5	ns	0.8904
30s:Control vs. 30s:AP5	*	0.0214

Area Under the Curve of arteriole dilation		
ANOVA table	F (DFn, DFd)	P value
Interaction	F (1, 28) = 11.83	P=0.0018
Stimulation length	F (1, 28) = 8.388	P=0.0072
AP5	F (1, 28) = 21.21	P<0.0001
Tukey's multiple comparisons test	Summary	Adjusted P Value
5s:Control vs. 5s:AP5	ns	0.8427
5s:Control vs. 30s:Control	***	0.0006
5s:Control vs. 30s:AP5	ns	0.6267
5s:AP5 vs. 30s:Control	****	<0.0001
5s:AP5 vs. 30s:AP5	ns	0.9803
30s:Control vs. 30s:AP5	****	<0.0001

Figure 5h

Peak neuropil Ca^{2+}		
ANOVA table	F (DFn, DFd)	P value
Interaction	F (1, 66) = 0.006692	P=0.9350
Stimulation length	F (1, 66) = 2.515	P=0.1175
AP5	F (1, 66) = 15.73	P=0.0002
Tukey's multiple comparisons test	Summary	Adjusted P Value
5s:Control vs. 5s:AP5	*	0.0464
5s:Control vs. 30s:Control	ns	0.6923
5s:Control vs. 30s:AP5	***	0.0009
5s:AP5 vs. 30s:Control	ns	0.3563
5s:AP5 vs. 30s:AP5	ns	0.6633
30s:Control vs. 30s:AP5	*	0.022

Area Under the Curve of neuropil Ca^{2+}		
ANOVA table	F (DFn, DFd)	P value
Interaction	F (1, 19) = 5.110	P=0.0357
Stimulation length	F (1, 19) = 11.54	P=0.0030
AP5	F (1, 19) = 7.358	P=0.0138
Tukey's multiple comparisons test	Summary	Adjusted P Value
5s:Control vs. 5s:AP5	ns	0.9891
5s:Control vs. 30s:Control	**	0.0031
5s:Control vs. 30s:AP5	ns	0.9591
5s:AP5 vs. 30s:Control	**	0.0024
5s:AP5 vs. 30s:AP5	ns	0.8603
30s:Control vs. 30s:AP5	**	0.0094

Supplementary Table 6: Details of statistical analysis related to Figure 6.

Figure 6c

**Peak arteriole dilation**		
ANOVA table	F (DFn, DFd)	P value
Interaction	F (1, 36) = 1.839	P=0.1835
Stimulation length	F (1, 36) = 1.325	P=0.2572
MSPPOH	F (1, 36) = 7.568	P=0.0092
Tukey's multiple comparisons test	Summary	Adjusted P Value
5s:Control vs. 5s:MSPPOH	ns	0.7581
5s:Control vs. 30s:Control	ns	0.3027
5s:Control vs. 30s:MSPPOH	ns	0.673
5s:MSPPOH vs. 30s:Control	*	0.0429
5s:MSPPOH vs. 30s:MSPPOH	ns	0.9989
30s:Control vs. 30s:MSPPOH	*	0.0304

Figure 6d

**Area Under the Curve of arteriole dilation**		
ANOVA table	F (DFn, DFd)	P value
Interaction	F (1, 36) = 5.522	P=0.0244
Stimulation length	F (1, 36) = 17.87	P=0.0002
MSPPOH	F (1, 36) = 9.268	P=0.0043
Tukey's multiple comparisons test	Summary	Adjusted P Value
5s:Control vs. 5s:MSPPOH	ns	0.9606
5s:Control vs. 30s:Control	***	0.0002
5s:Control vs. 30s:MSPPOH	ns	0.8366
5s:MSPPOH vs. 30s:Control	****	<0.0001
5s:MSPPOH vs. 30s:MSPPOH	ns	0.5517
30s:Control vs. 30s:MSPPOH	**	0.0028
