# Supplementary material for: Astrocytes amplify neurovascular coupling to sustained activation of neocortex in awake mice
Source: Nat Commun. 2022 Dec 22;13:7872. doi: 10.1038/s41467-022-35383-2 (PMC9780254; doi:10.1038/s41467-022-35383-2)
Supplement: Supplementary file 3 — Reporting Summary [file 41467_2022_35383_MOESM3_ESM.pdf]

nature portfolio

Corresponding author(s): Grant R Gordon

Last updated by author(s): Nov 4, 2022

Reporting Summary

Nature Portfolio wishes to improve the reproducibility of the work that we publish. This form provides structure for consistency and transparency in reporting. For further information on Nature Portfolio policies, see our Editorial Policies and the Editorial Policy Checklist.

Statistics

For all statistical analyses, confirm that the following items are present in the figure legend, table legend, main text, or Methods section.

n/a	Confirmed	
☐	☒	The exact sample size (n) for each experimental group/condition, given as a discrete number and unit of measurement
☐	☒	A statement on whether measurements were taken from distinct samples or whether the same sample was measured repeatedly
☐	☒	The statistical test(s) used AND whether they are one- or two-sided<br>*Only common tests should be described solely by name; describe more complex techniques in the Methods section.*
☐	☒	A description of all covariates tested
☐	☒	A description of any assumptions or corrections, such as tests of normality and adjustment for multiple comparisons
☐	☒	A full description of the statistical parameters including central tendency (e.g. means) or other basic estimates (e.g. regression coefficient) AND variation (e.g. standard deviation) or associated estimates of uncertainty (e.g. confidence intervals)
☐	☒	For null hypothesis testing, the test statistic (e.g. F, t, r) with confidence intervals, effect sizes, degrees of freedom and P value noted<br>*Give P values as exact values whenever suitable.*
☒	☐	For Bayesian analysis, information on the choice of priors and Markov chain Monte Carlo settings
☒	☐	For hierarchical and complex designs, identification of the appropriate level for tests and full reporting of outcomes
☒	☐	Estimates of effect sizes (e.g. Cohen's d, Pearson's r), indicating how they were calculated

Our web collection on statistics for biologists contains articles on many of the points above.

Software and code

Policy information about availability of computer code

Data collection	Two-photon imaging data were collected with open source Scan Image aquisition software Version 3.81. Behavioral recordings were collected with Pylon Viewer Version 6.2.4.9387 by Basler. Intrinsic optical signals were recorded through Pylon Viewer Version 5.0.11.10913 by Basler.
Data analysis	Data were analyzed with ImageJ Version 2.9.0 (NIH), Prism Version 8.2 (Graph Pad Software, LLC), Amazon Mechanical Turk: Vessel Annotation Task (http://doi.org/10.5281/zenodo.7226673), and MATLAB and Statistics Toolbox Release 2019b (The MathWorks, Inc., Natick, MA, USA) (https://senselab.med.yale.edu/modeldb/ShowModel?model=266929#tabs-l) and MATLAB and Statistics Toolbox Release 2021b.<br><br>Calcium imaging data were analyzed with ImageJ Version 2.9.0 (NIH), and with the MATLAB-based Astrocyte Quantification and Analysis (AQuA) (https://github.com/yu-lab-vt/AQuA) script.<br><br>Locomotion recordings were analyzed with MATLAB and Statistics Toolbox Release 2021b (https://github.com/tpgovind/Locomotion-Pupil-Tracking/blob/main/locomotion_trace_script.m)(DOI:10.5281/zenodo.7279708), and the Pupil diameter tracking tool (http://doi.org/10.5281/zenodo.7246747 (2022) with DeepLabCut (https://github.com/DeepLabCut/DeepLabCut).

For manuscripts utilizing custom algorithms or software that are central to the research but not yet described in published literature, software must be made available to editors and reviewers. We strongly encourage code deposition in a community repository (e.g. GitHub). See the Nature Portfolio guidelines for submitting code & software for further information.

Data

Policy information about availability of data

All manuscripts must include a data availability statement. This statement should provide the following information, where applicable:

- Accession codes, unique identifiers, or web links for publicly available datasets
- A description of any restrictions on data availability
- For clinical datasets or third party data, please ensure that the statement adheres to our policy

Source data are provided with this paper. Raw image files are stored on servers at Hotchkiss Brain Institute owing to their large size. These raw images can be provided from the corresponding author upon request.

Human research participants

Policy information about studies involving human research participants and Sex and Gender in Research.

Reporting on sex and gender	n/a
Population characteristics	n/a
Recruitment	n/a
Ethics oversight	n/a

Note that full information on the approval of the study protocol must also be provided in the manuscript.

Field-specific reporting

Please select the one below that is the best fit for your research. If you are not sure, read the appropriate sections before making your selection.

☒ Life sciences ☐ Behavioural & social sciences ☐ Ecological, evolutionary & environmental sciences

For a reference copy of the document with all sections, see nature.com/documents/nr-reporting-summary-flat.pdf

Life sciences study design

All studies must disclose on these points even when the disclosure is negative.

Sample size	Based on previous effect sizes and error, n of 5-10 is typically sufficient power to test for significance in brain slices (Institoris, A. et al., J. Cereb. Blood Flow Metab. 35, 1411-1415 (2015); Rosenegger et al., J. Neurosci. 35, 13463–13474 (2015). For in vivo observational experiments (Fig. 1-3, Supplementary Fig. 1-3 and 7-8) we expected to be able to collect data from 2 vessels per mouse on average, based on the time period an awake mouse tolerates the experiment.
Data exclusions	No data were excluded.
Replication	In Figure 1E, astrocyte endfoot and process Ca2+ and arteriole dilation response latencies match the findings described in a previous paper by the Gordon lab, using an identical but different 2-photon fluorescent microscopy rig. See Tran, C.H. et al., Neuron 100, 1133-1148.e3 (2018).<br><br>Astrocyte process Ca2+ signals of Aldh1l1-cre x RCL-lck-GCaMP6f mice (Supplementary Fig. 2) matches the observation by Stobart et al., Neuron 98(4):726-735.e4. doi: 10.1016/j.neuron.2018.03.050 (2018).<br><br>The effectiveness of the AAV2/5-gfaABC1D-CalEx-mCherry construct in reducing astrocyte Ca2+ signaling has been described in details in Yu, X. et al., Neuron 99, 1170-1187.e9 (2018).<br><br>In the previous submission of the manuscript the CalEx experiments were carried out using an acute cranial window preparation and a less optimal viral control, astrocyte targeted tdTomato. The main effect of CalEx reducing the late phase of arteriole dilation to 30sec whisker stimulation was the same. However, in astrocytes expressing tdTomato, the measured astrocyte Ca2+ signals were reduced, most likely as a result of tdTomato quenching the fluorescent light emitted by GCaMP. This rendered the effect of CalEx on attenuating astrocyte Ca2+ to be less significant than in the published CalEx experiment.<br><br>The novel kinetics of penetrating arteriole responses has been reproduced by our lab using intrinsic optical imaging, which is available as a pre-print on BioRXiv (doi: https://doi.org/10.1101/2021.09.15.460557).<br><br>Vascular and cellular Ca2+ responses of short electrical stimulation of rat brain slices have been previously identified and reliably reproduced in Institoris, A. et al., J. Cereb. Blood Flow Metab. 35, 1411-1415 (2015).

Randomization	Randomization was not applicable because in the majority of experiments within subject comparison was done (pre-drug vs post-drug). Between subject comparison was only used in the CalEx experiments. We randomly picked which mouse would receive CalEx or Control virus injection on the day of the surgery. We ran the same imaging experimental protocol.
Blinding	MATLAB and ImageJ analysis of vessel diameter, astrocyte calcium and the analysis of event-based neuronal calcium in the in vivo CalEx experiments were blinded. Vessel diameter of astrocyte Gq-DREADD and MSPPOH in vivo experiments were outsourced to Amazon Mechanical Turk users.

Reporting for specific materials, systems and methods

We require information from authors about some types of materials, experimental systems and methods used in many studies. Here, indicate whether each material, system or method listed is relevant to your study. If you are not sure if a list item applies to your research, read the appropriate section before selecting a response.

Materials & experimental systems

n/a	Involved in the study
☐	☒ Antibodies
☐	☒ Eukaryotic cell lines
☒	☐ Palaeontology and archaeology
☐	☒ Animals and other organisms
☒	☐ Clinical data
☒	☐ Dual use research of concern

Methods

n/a	Involved in the study
☒	☐ ChIP-seq
☒	☐ Flow cytometry
☒	☐ MRI-based neuroimaging

Antibodies

Antibodies used	Anti-Hemagglutinin (rabbit) primary antibody (1:100) from Abeam (Ab9110).
Validation	Anti-Hemagglutinin (rabbit) primary antibody (1:100: Abcam) has been previously validated in a large body of publications, including Perreten Lambert H et al. Control of mitochondrial pH by uncoupling protein 4 in astrocytes promotes neuronal survival. J Biol Chem 289:31014-28 (2014).

Eukaryotic cell lines

Policy information about cell lines and Sex and Gender in Research

Cell line source(s)	ThermoFisher Scientific
Authentication	The cell line 293FT used in the study was not authenticated.
Mycoplasma contamination	Cell lines were not tested for mycoplasma contamination. There is an annual mycoplasma testing of all the cell lines which has been negative.
Commonly misidentified lines (See ICLAC register)	No misidentified cell lines were used in the study.

Animals and other research organisms

Policy information about studies involving animals; ARRIVE guidelines recommended for reporting animal research, and Sex and Gender in Research

Laboratory animals	Mice were male: P22-90 c57bl/6 mice (n=41: 5 acute window; 11 Cal Ex; 11 mutant Cal Ex control, 4 MSPPOH; 7 AP5; 3 thinned skull), transgenic Aldh1l1-Cre/ERT2 x RCL-GCaMP6s (Ai96)(n=9), Aldh1l1-Cre/ERT2 x R26-Lck-GCaMP6f (n=13), PDGFRB-Cre x RCL-GCaMP6s (n=11), Aldh1l1-Cre/ERT2 x CAG-LSL-Gq-DREADD (n=11), C57BL/6J-Tg(Thyl-GCaMP6f) (n=6).<br>Sprague-Dawley rats, male, P21-33.
Wild animals	No wild animals were used in the study.
Reporting on sex	All mice were male.
Field-collected samples	No field collected samples were used in the study.
Ethics oversight	All animal procedures were performed in accordance with guidelines approved by the Animal Care and Use committee of the University of Calgary (protocols AC19-0170 and AC19-0109).

Note that full information on the approval of the study protocol must also be provided in the manuscript.
